# Lysozyme Associated Liposomal Gentamicin Inhibits Bacterial Biofilm

**DOI:** 10.3390/ijms18040784

**Published:** 2017-04-09

**Authors:** Yilin Hou, Zhaojie Wang, Peng Zhang, Hu Bai, Yuelin Sun, Jinyou Duan, Haibo Mu

**Affiliations:** Shaanxi Key Laboratory of Natural Products & Chemical Biology, College of Chemistry & Pharmacy, Northwest A&F University, Yangling 712100, China; stellanox1019@163.com (Y.H.); wzhj10111@nwafu.edu.cn (Z.W.); 15291850570@163.com (P.Z.); baihu@nwafu.edu.cn (H.B.); sunyuelin_123@nwafu.edu.cn (Y.S.); jduan@nwafu.edu.cn (J.D.)

**Keywords:** liposome, lysozyme, gentamicin, biofilm

## Abstract

Bacteria on living or inert surfaces usually form biofilms which make them highly resistant to antibiotics and immune clearance. Herein, we develop a simple approach to overcome the above conundrum through lysozyme-associated liposomal gentamicin (LLG). The association of lysozyme to the surface of liposomes can effectively reduce the fusion of liposomes and undesirable payload release in regular storage or physiological environments. The LLG was more effective at damaging established biofilms and inhibiting biofilm formation of pathogens including Gram-positive and Gram-negative bacteria than gentamicin alone. This strategy may provide a novel approach to treat infections due to bacterial biofilm.

## 1. Introduction

Biofilms are matrix-enclosed, complex and differentiated communities of bacteria that are adherent to inert or biological surfaces [1]. Upon adhesion, the bacterial cells start producing an extracellular matrix and group together in densely-packed bacterial clusters. From the mature biofilm, individual cells or biofilm fragments are released and can colonize new surfaces [2]. Biofilm formation causes corrosion and biofouling of industrial equipment [3], and is also associated with many illnesses and infections in humans, including oral diseases, native valve endocarditis, and a number of nosocomial infections [2]. Unlike planktonic bacteria, biofilm bacteria have been known to be highly resistant to adverse environmental conditions such as antibiotics, detergents or biocides [4]. Therefore, new efforts for biofilm growth inhibition, biofilm damage, or biofilm eradication are being sought. 

The biggest challenge in treating biofilm infections is overcoming the resistance and tolerance to antimicrobial agents. Successful therapy requires innovative ways to deliver antimicrobial substances in a sufficiently high concentration to the biofilm bacteria [5]. Liposome is an attractive candidate for drug delivery to biofilms due to its versatility and biocompatibility. It has been shown that liposome-encapsulation improves the efficacy of various antibacterial and antifungal drugs against a broad range of pathogens in vitro and in vivo [6,7,8,9]. One advantage of liposomes being used as drug delivery vehicles is their potential to fuse with phospholipid membranes. However, the applications of liposomes, particularly those with sizes below 100 nm, are often hindered by their poor stability due to spontaneous fusion, resulting in payload loss and increase in vesicle size [9,10]. Several strategies have been employed to overcome this problem including optimizing liposome composition [11], polyethylene glycol (PEG)ylation [12] and nanoparticulate stabilizing [13,14,15]. 

Herein, we introduce a novel liposome formulation stabilized by lysozyme. The cationic lysozyme is ready to associate with the negatively charged liposome through electrostatic attraction. This strategy stabilizes liposomes against fusion and avoids undesirable leakage of liposomal drugs. The stabilized liposomes with positive charge are hypothesized to easily bind to the negatively charged matrix of bacterial biofilm, inhibit bacterial biofilm formation and damage established biofilm built by Gram-positive or -negative organisms. 

## 2. Results and Discussions

### 2.1. Preparation and Characterization of Lysozyme-Associated Liposomal Gentamicin

**Figure 1:**
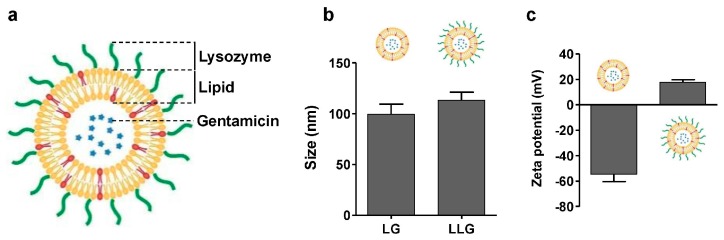
(**a**) Schematic structure of lysozyme-associated liposomal gentamycin (LLG); (**b**) hydrodynamic size; and (**c**) surface zeta potential of liposome (without Lysozyme) and LLG.

Liposomal gentamicin (LG) was prepared by vesicle extrusion technique (Figure 1a) [16]. The size and surface zeta potential of LG were 99 nm and −54.5 mV, respectively (Figure 1b,c). Lysozyme-associated liposomal gentamicin (LLG) were obtained by mixing LG and lysozyme. The size and surface zeta potential of the resulting LLG were measured by dynamic light scattering (DLS). The size of LLG was slightly larger than that of LG, suggesting the adsorption of lysozyme onto the liposomal surface. The surface zeta potential changed from −54.5 to 17.5 mV (Figure 1c), which confirmed the association of positively-charged lysozyme to the negatively-charged liposomes through electrostatic attraction.

### 2.2. Stability of Lysozyme-Associated Liposomal Gentamicin

**Figure 2:**
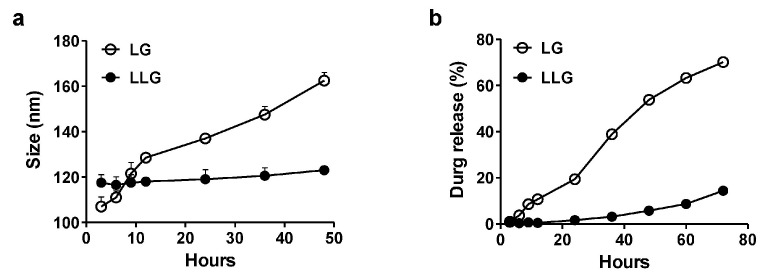
(**a**) Size measurements of liposome and LLG over the course of 48 h at 25 °C in deionized water; (**b**) cumulative release profile of gentamicin-loaded liposome and LLG over the course of 72 h at 25 °C in deionized water.

The stability of LG and LLG were evaluated over time in deionized water (Figure 2a). LG was gradually aggregated while LLG was relatively stable in water. Gentamicin in LG was released more quickly than in LLG (Figure 2b). These data suggested that the association of lysozyme improved the stability of liposome and prevented the release of gentamicin.

### 2.3. Antibiofilm Activities of Lysozyme-Associated Liposomal Gentamicin

**Figure 3:**
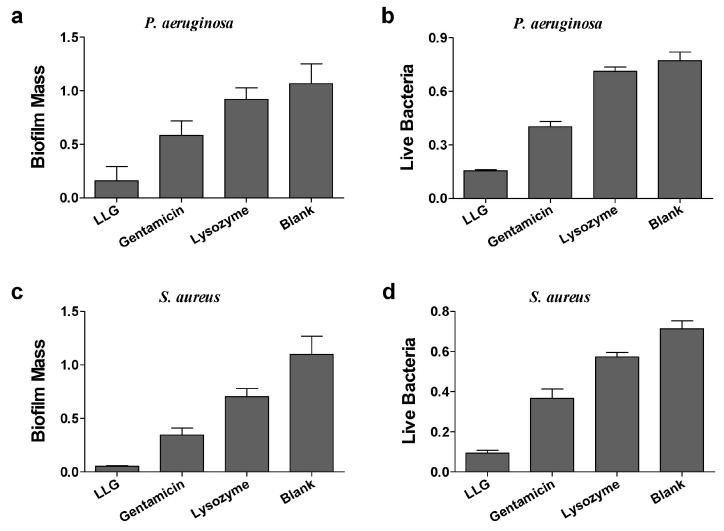
Crystal violet assay and 3-(4,5-dimethyl-thiazol-2-yl)-2,5-diphenyltetrazolium bromide (MTT) assay to assess the antibiofilm activity of LLG against *P. aeruginosa* biofilm (**a**,**b**) and *S. aureus* (**c**,**d**).

*Pseudomonas aeruginosa* (*P. aeruginosa*) is a Gram-negative opportunistic human pathogen, which is known for causing chronic pulmonary infections in cystic fibrosis (CF) patients and patients suffering of non-CF bronchiectasis, and generally employed as a model organism for investigation of biofilms [17]. Gentamicin or lysozyme alone had a mild effect on biomass and live cells of *P. aeruginosa* biofilms after 24 h treatment compared to blank control (Figure 3a,b). LLG treatment markedly reduced both biofilm mass and viable cell counts. To see whether LLG was able to eliminate bacterial biofilms built by a Gram-positive organism, *Staphylococcus aureus* (*S. aureus*), which can cause life-threatening infections in humans and the nosocomial (hospital) environment [18], was tested. Quantification of biofilm biomass and cell viability demonstrated that LLG had a more pronounced effect than gentamicin or lysozyme alone (Figure 3c,d).

**Figure 4:**
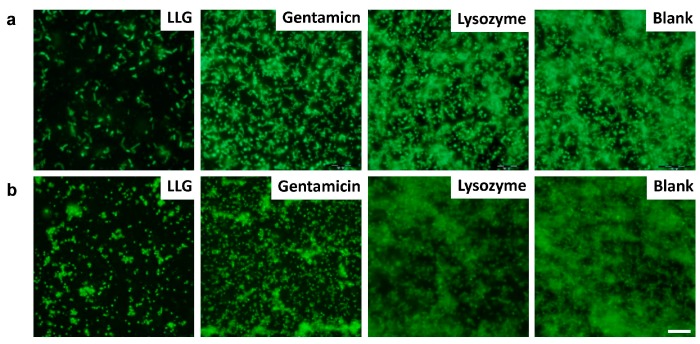
Fluorescence microscopy of *P. aeruginosa* (**a**) and *S. aureus* (**b**) biofilm. Biofilms incubated with tryptic soy broth (TSB) are used as control. Scale bars were 10 μm.

Fluorescence microscopy imaging of *P. aeruginosa* (Figure 4a) and *S. aureus* (Figure 4b) biofilms was pursued to further evaluate the antibiofilm potential of LLG. The blank control biofilms were densely colonized with hierarchically and three-dimensionally structured formations. Biofilms treated with LLG exhibited only a few isolated bacterial colonies instead of a recognizable biofilm structure. 

**Figure 5:**
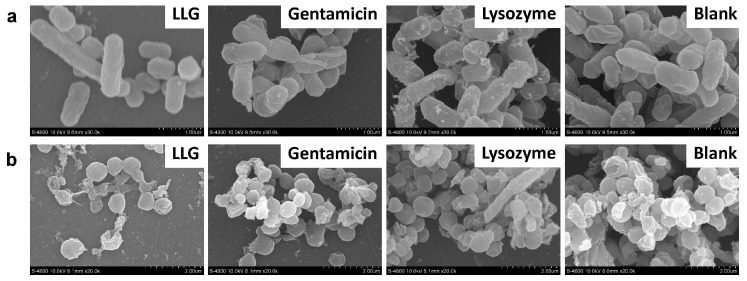
Scanning electron microscopy of *P. aeruginosa* (**a**) and *S. aureus* (**b**) biofilm. Biofilms incubated with TSB are used as control. Scale bars were 1 μm.

Scanning electron microscopy (SEM) was also applied to evaluate the surface morphology changes of *P. aeruginosa* (rod shaped pathogen, Figure 5a) and *S. aureus* (round-shaped pathogen, Figure 5b) biofilms treated with LLG, gentamicin or lysozyme in tryptic soy broth (TSB). The control showed a highly organized and well-defined architecture. In LLG-treated biofilms, the cell walls became wrinkled and damaged, the shape and size of cells changed dramatically, and only a few scattered bacterial cells were noted. Overall, these results clearly indicated that LLG had an advantage in disrupting existing biofilms formed by Gram-negative and -positive organisms. 

**Figure 6:**
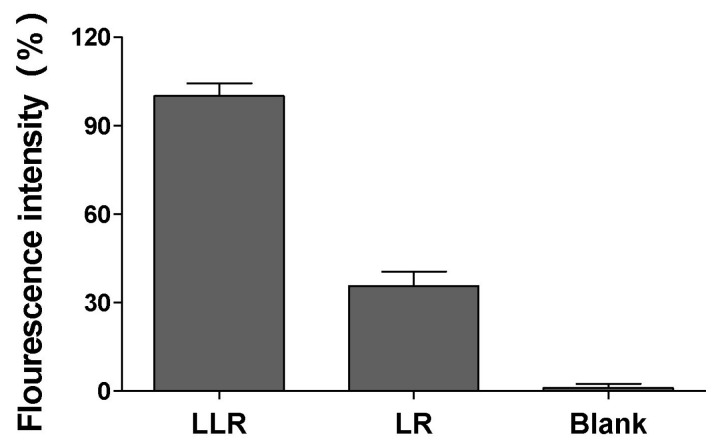
Binding ability of lysozyme liposome to *S. aureus* biofilm. Lysozyme associated liposomal rhodamine B (LLR) or liposomal rhodamine B (LR) was incubated with preformed *S. aureus* biofilm for 10 min. After incubation, the biofilm was collected and quantified for fluorescence intensity. The biofilm without incubating with any liposome formulations was tested in parallel serving as the background signal.

To explore the underlying mechanism by which LLG disrupted bacterial biofilms above, liposomal rhodamine B (LR) or lysozyme associated liposomal rhodamine B (LLR) were generated. Compared with LR, LLR elicited a much stronger binding to *S. aureus* biofilm (Figure 6). This might be due to the electrostatic attraction between positive lysozyme on LLR and biofilm matrix, such as alginate, which usually possesses a negative charge.

**Figure 7:**
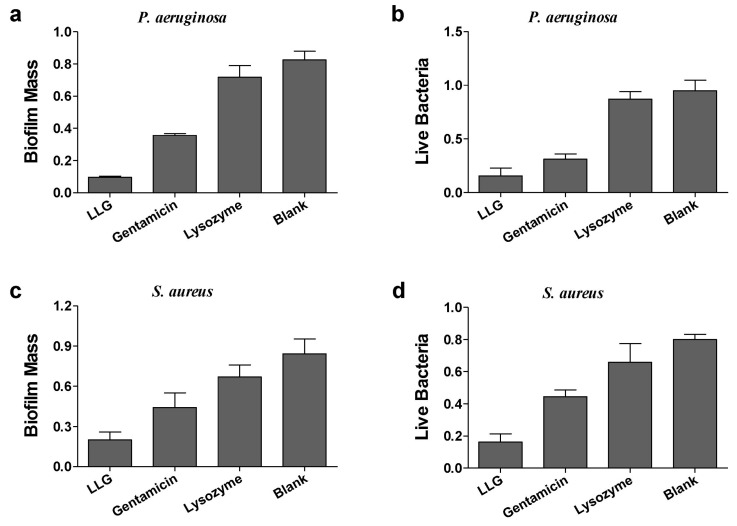
The inhibitory effects of LLG on *P. aeruginosa* (**a**,**b**) and *S. aureus* (**c**,**d**) biofilm formation.

Biofilm formation was examined in the case of planktonic *P. aeruginosa* (Figure 7a,b) exposed to LLG, gentamicin or lysozyme for 24 h. Lysozyme showed no effects on biofilm formation as compared with blank control. Gentamicin suppressed biofilm formation and decreased live cells generally whereas LLG facilitated this suppression and reduction significantly. Similar findings were also observed in the case of *S. aureus* by quantification of biofilm biomass (Figure 7c) and cell viability (Figure 7d). These results suggested that LLG had the potential to prevent planktonic cells of Gram-negative or -positive organisms from biofilm formation.

Controlled drug delivery by lipid nanoparticles have attracted much attention. More recently, Harker et al. utilized an electrohydrodynamic technique to prepare core-shell lipid nanoparticles with a tunable size and high active ingredient loading capacity, encapsulation efficiency and controlled release [19]. Many nano materials, for example, the metal-loaded nanofibers [20,21,22], have shown high antibacterial activity against planktonic or biofilm bacteria [23,24]. In the current study, we developed a platform to deliver antibiotic to treat bacterial biofilms through lysosome associated liposomes. This approach made liposomes more stable and easier to attach to biofilms; a universal survival lifestyle for microbes in nature.

## 3. Materials and Methods

### 3.1. Materials

1,2-dipalmitoyl-*sn*-glycero-3-phosphocholine (DPPC) and 1,2-dipalmitoyl-*sn*-glycero-3-phosphor-(1′-rac-glycerol) (DPPG) were purchased from Avanti Polar Lipids (Alabaster, AL, USA). Gentamicin Sulfate was purchased from Solarbio (Beijing, China). Rhodamine B was purchased from Aladdin (Shanghai, China). All reagents were of analytical grade and used as received without further purifying.

*Pseudomonas aeruginosa* (PAO1) and *Staphylococcus aureus* (ATCC 29213) were generous gifts received from Xiaodong Xia (College of Food Science and Engineering, Northwest A&F University).

### 3.2. Preparation and Characterization of LLG

Liposomes were prepared following a previously described extrusion method [16]. Briefly, 9 mg of lipid (DPPC/DPPG = 9/1, molar ratio) were dissolved in 1 mL chloroform, and then the organic solvent was evaporated to form a dried lipid film. The lipid film was rehydrated with 3 mL of deionized water, or 2 mM rhodamine B (RhB), or 20 mM gentamicin, followed by vortexing for 1 min and sonicating for 5 min to produce multilamellar vesicles (MLVs). The solution was extruded through a 100 nm pore-sized polycarbonate membrane for 10 times to form narrowly distributed small unilamellar vesicles (SUVs). Particles were purified by washing with water 3 times using 10 kDa MWCO Amicon centrifugal filters (EMD Millipore, Billerica, CA, USA) to remove unencapsulated drugs. To prepare LLG, the pH of both lysozyme and liposome solutions was adjusted to 6.5 using HCl. Then the liposomes and lysozyme at 1:100 (molar ratio) were mixed together, followed by 10 min bath sonication. The hydrodynamic size and surface zeta potential of LLG were measured by dynamic light scattering (DLS) measurements (Malvern Zetasizer NANO-ZS90, Malvern, UK). The gentamicin content was determined by sodium phosphotungstate precipitation method.

### 3.3. Stability Studies

Stability of LLG or bare liposome was analyzed in deionized water. 2 mL freshly prepared liposome samples at 1 mg·mL^−1^ were incubated at 25 °C for 48 h. The size change of the liposome samples in deionized water were measured by DLS.

### 3.4. Release Behaviors

The kinetics of gentamicin release was studied from the prepared LLG. The 1 mL fresh prepared liposome solution (2 mg·mL^−1^) was initially incubated at 25 °C in tube. Liposomes were taken at regular time intervals, centrifuged using 10 kDa MWCO Amicon centrifugal filters (EMD Millipore, Billerica, CA, USA) and the filtrate was obtained for gentamicin measurement. 

### 3.5. Binding Ability of Lysozyme Liposome to Biofilm

Lysozyme associated liposomal RhB (LLR) and liposomal RhB (LR) were prepared as described in Section 3.2. *S. aureus* (~10^9^ colony forming units (CFU)) were grown in 6-well plates at 37 °C for 24 h supplemented with 2 mL of TSB to allow biofilm formation. The non-adhered cells were removed with pipette and the plate was washed three times using 0.9% (*w*/*v*) NaCl. Then existing biofilms were incubated at 37 °C in 1.8 mL TSB supplemented with 0.2 mL LLR or LR for 10 min. After that, the medium was removed and the biofilm was washed, collected using cell scraper in phosphate buffered saline (PBS), vortexed and subjected to fluorescence detection on a RF-5301 fluorescence spectrometer (Shimadzu, Kyoto, Japan) at excitation and emission wavelengths of 509 nm and 526 nm, respectively. Relative fluorescence intensity was expressed as percentage, and biofilm treated with LLG was used as 100% fluorescence intensity.

### 3.6. Antibiofilm Activity

As described previously [25], 100 μL bacterial TSB solutions (~10^8^ CFU) were seeded into 96-well polystyrene microtitre plates (Corning, NY, USA) at 37 °C for 24 h to allow biofilm formation. The non-adhered cells were removed with pipette and the plate was washed three times using 100 μL 0.9% (*w*/*v*) NaCl. Then existing biofilms were incubated at 37 °C in 90 μL TSB supplemented with 10 μL LLG, equivalent gentamicin or lysozyme for 24 h. Each treatment included 6 parallel wells. Biofilms incubated with TSB only were used as blank. Biofilm mass (crystal violet staining assay) and viable cells (MTT (3-(4,5-dimethyl-thiazol-2-yl)-2,5-diphenyltetrazolium bromide) assay) were evaluated as previous [26]. All experiments were performed 3 times. Error bars represent standard deviation (SD). 

For biofilm inhibition assay, 100 μL of bacteria in TSB (~10^8^ CFU) were seeded into individual wells of microtiter plates in the presence of compounds for 24 h. Biofilm mass were evaluated as described [26].

For fluorescence microscopy, *S. aureus* or *P. aeruginosa* (~10^8^ CFU) was grown on glass coverslips at 37 °C for 24 h in 24-well plates supplemented with 1 mL of TSB to allow biofilm formation. The coverslips were washed to remove unattached cells and were treated with liposomes for 24 h at 37 °C. Existing biofilms were treated and imaged as previous [25]. SEM was conducted as described previously [27].

### 3.7. Statistical Analysis

All graphical evaluations were made using GraphPad Prism 5.0 (GraphPad Software Inc., San Diego, CA, USA). Analysis of variance (ANOVA) was used to evaluate significant differences.

## 4. Conclusions

In this study, we applied positively-charged lysozyme to stabilize the negatively-charged liposomes through electrostatic attraction. The lysozyme-associated liposomal gentamycin (LLG) was more effective at disrupting the preformed biofilms built by Gram-positive and -negative pathogenic bacteria than lysozyme or gentamycin. Further study demonstrated that lysozyme associated liposomes could attach to the biofilm matrix, such as alginate, which usually possessed a negative charge. Meanwhile, LLG was shown to prevent planktonic bacterial cells from biofilm formation. This strategy provided a novel platform for antibiotic delivery and might be useful to develop new therapeutics for treatment of chronic and stubborn infections related to microbial biofilm.
